# Thousands of domestic and public supply wells face failure despite groundwater sustainability reform in California’s Central Valley

**DOI:** 10.1038/s41598-023-41379-9

**Published:** 2023-09-08

**Authors:** Darcy Bostic, Linda Mendez-Barrientos, Rich Pauloo, Kristin Dobbin, Victoria MacClements

**Affiliations:** 1 Rural Community Assistance Corporation, West Sacramento, USA; 2 Josef Korbel School of International Studies, University of Denver, Denver, USA; 3 Water Data Lab, Berkeley, USA; 4 Department of Environmental Science, Policy, and Management, University of California Berkeley, Berkeley, USA

**Keywords:** Sustainability, Environmental sciences, Hydrology

## Abstract

Across the world, declining groundwater levels cause wells to run dry, increase water and food insecurity, and often acutely impact groundwater-dependent communities. Despite the ubiquity and severity of these impacts, groundwater research has primarily focused on economic policy instruments for sustainable management or the quantification of groundwater depletion, rather than assessing the impacts of management decisions. In particular, how definitions of groundwater sustainability shape the fate of resource users remains unexplored. Here, we examine one of the world’s largest-scale environmental sustainability reforms, the California Sustainable Groundwater Management Act (SGMA), and estimate the impact of sustainability definitions proposed in groundwater sustainability plans (GSPs) on well failure. We show that locally-proposed sustainability criteria are consistent with business as usual groundwater level decline, and if reached, could impact over 9000 domestic wells and around 1000 public supply wells. These findings highlight the necessity of careful and critical evaluation of locally-developed sustainability definitions and their implementation to prevent detrimental impacts, such as threats to household and municipal water supply.

## Introduction

Groundwater level decline causes wells to run dry^1^, poses threats to water and food security^2,3^, tends to acutely impact low-income communities of color^4^, and causes other well-known consequences (e.g., land subsidence^5^, deterioration of groundwater storage and quality^6,7^). Although the consequences of groundwater depletion in key food-producing regions of the world are well understood^8^, sustainable groundwater research has mainly concentrated on economic policy instruments (e.g., property rights, markets, subsidies and/or taxes^9^) to curb groundwater decline or addressed the “we can’t manage what we can’t measure” paradigm^10^ by quantifying groundwater depletion^11,12^ and well vulnerability (e.g.,. to drought^13^ or contaminants^14^^)^. These studies are predominantly concerned with the consequences of unsustainable groundwater management, but do not evaluate how definitions of groundwater sustainability created by local, decentralized, planning efforts shape the implementation of groundwater sustainability reforms and their aggregate outcomes across large, interconnected hydrologic regions.

Understanding the impact of varying local definitions of sustainability in natural resource management reforms is critical given the growing preference for decentralized, polycentric approaches to natural resource management^15,16^. Under this type of reform, local resource users organize to establish decentralized governance agencies and develop management plans^17^, while coordinating with other actors and organizations at multiple scales^18^. The promise of polycentrism to solve multiple environmental governance challenges has been widely highlighted, gaining recognition as an effective approach to integrate the voices of resource users and manage natural resources^19^. However, like all sustainability reforms, reforms following a polycentric approach are inherently political^20,21^ and often face pressure from incumbent regimes and powerful interests to set less stringent goals^22^. As a result, disproportionate negative impacts on resource users with less financial and political power is possible^23^.

Groundwater governance is a quintessential example of the challenge of polycentric environmental governance^10^. Here, we examine the California Sustainable Groundwater Management Act (SGMA), one of the world’s few large-scale environmental reforms that aims to implement a polycentric governance system to manage groundwater resources sustainably^24^. SGMA broadly defines sustainability as “the management and use of groundwater in a manner that can be maintained during the planning and implementation horizon without causing undesirable results,”^25^ and leaves local actors to decide what type of governance arrangements and rules best achieve these goals. Local actors are allowed to self-organize into groundwater sustainability agencies (GSAs), which are tasked with the development and implementation of groundwater sustainability plans (GSPs). These GSAs must set their sustainable management criteria within GSPs, including defining undesirable results to be avoided, minimum thresholds, and measurable objectives. Undesirable results include six physical effects, including among others, the chronic lowering of groundwater levels. The success of SGMA largely depends on how local actors implement these sustainability criteria as described in the Act and in the Best Management Practices developed by the California Department of Water Resources (DWR).

In this paper, we specifically examine minimum thresholds (MTs) because they represent the minimum acceptable groundwater elevation, defined at discrete locations (i.e., monitoring wells) to avoid undesirable results. In other words, MTs are the limits between conditions deemed locally acceptable, and those deemed unacceptable on the basis of their impacts to groundwater users. Each GSP defines MTs at monitoring wells spread across the jurisdictional area at a spatial intensity that aims to sample groundwater conditions and potential impacts to beneficial users of groundwater in the region. By statute, MTs are set at groundwater levels that prevent undesirable impacts to all beneficial users of groundwater. In other words, they must balance the needs of agricultural, domestic, and environmental groundwater users. Furthermore these MTs, as well as the GSPs themselves, must be coordinated at the subbasin level, and are reviewed and approved by central state agencies. For these reasons, in this paper MTs act as the material representation of the definition of sustainability for each GSP.

We evaluate sustainability definitions in 60 groundwater sustainability plans (GSPs) within 35 subbasins in California’s Central Valley aquifer system, and estimate the impact of minimum thresholds (MTs) on the failure of drinking water wells. Specifically, because we cannot know if and when MTs will be reached by each basin individually, we model the hypothetical worst-case scenario that all MTs are simultaneously reached and calculate the resulting impact on drinking water wells, which are critical for household food and water security. We ask: (1) How deep are MT groundwater levels compared to a 2019 baseline and a 2040 business as usual projection? (2) What are the spatial impacts of MTs on drinking water wells (e.g., domestic and public supply)? (3) To what extent are vulnerable groups, such as disadvantaged communities (DACs), included in the monitoring and measurement of progress towards sustainability (e.g., via groundwater well monitoring networks that define MTs)?

## Results

### Groundwater levels are projected to decline under SGMA

**Figure 1:**
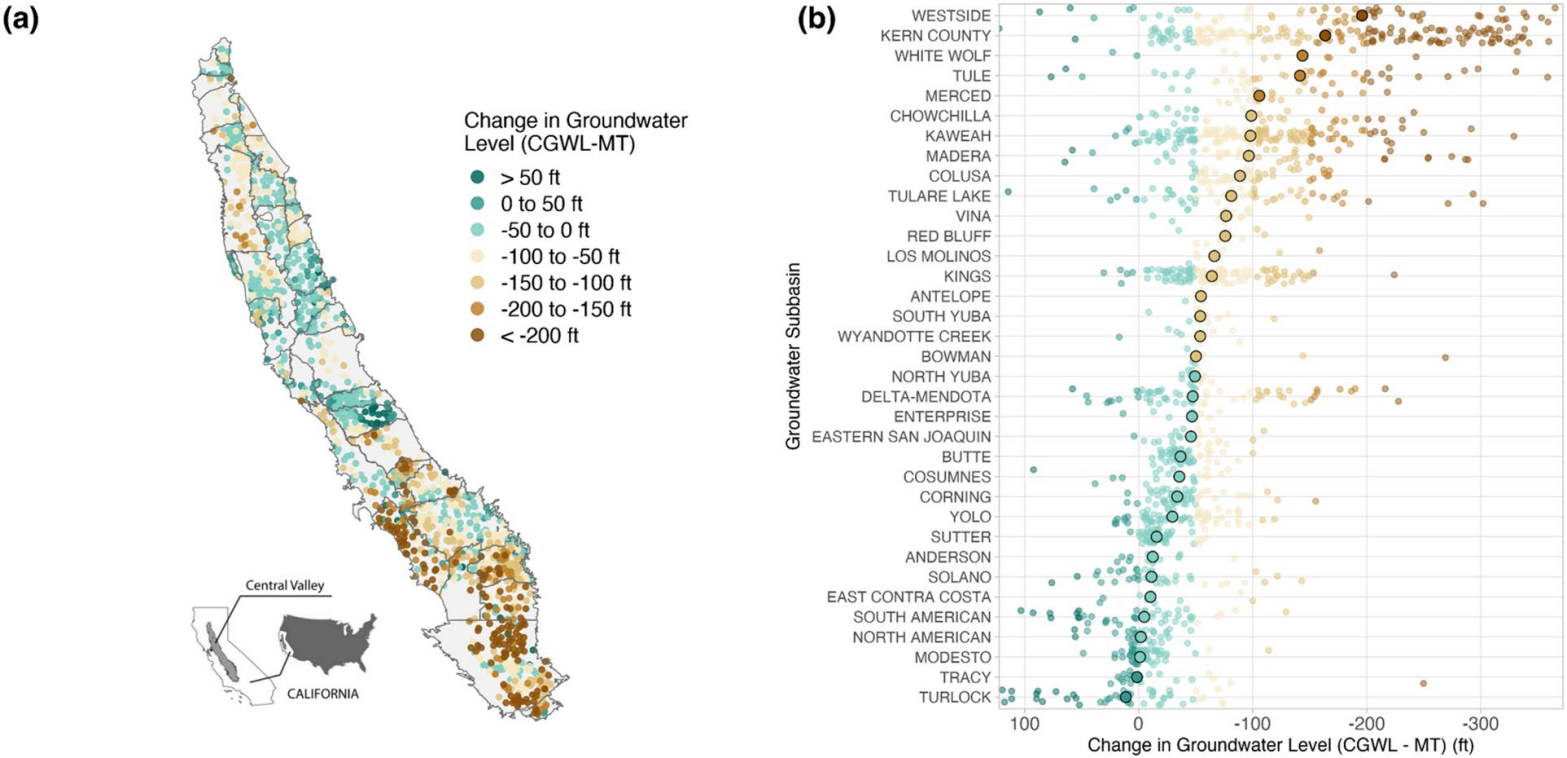
Estimated groundwater elevation changes (x-axis) per subbasin (y-axis) under proposed MTs. Points in subplots a-b represent a change in groundwater level between the CGWL in 2019 and the proposed MT. (**a**) Southern Central Valley basins generally set MTs lower than the 2019 CGWL compared to central and northern basins. (**b**) Differences between MTs and the 2019 CGWL are distributed between roughly + 100 feet (MT is higher than CGWL) and -350 feet (MT is lower than CGWL). Median changes between MTs and CGWL, shown as darkly circled points in subplot b, suggest generally between less than 100 feet of decline, but upwards of 200 feet of decline in some basins.

In all 35 subbasins evaluated, GSPs set a portion of MTs below current groundwater levels (Fig. 1), effectively permitting further groundwater depletion before MTs are reached. Averaged across all monitoring wells in all subbasins, MTs are nearly 80 feet deeper than a current groundwater level (CGWL) baseline derived from groundwater conditions in 2019. Moreover, 23 out of 60 GSPs, located in 13 of 35 subbasins, defined over 150 MTs (11% of all MTs) over 200 feet below the 2019 CGWL. Although many GSPs set MTs far below current groundwater elevations (e.g., tens of feet), some regions with generally higher groundwater levels, like the northern Central Valley, set MT ranges closer to present day levels.

### Groundwater level declines are consistent with business as usual extraction

MTs are set at levels that allow for continued groundwater level decline consistent with a business as usual (BAU) rate of decline. We compared the estimated MT groundwater surface from this study to a modeled BAU groundwater level from Pauloo et al. (2020), based on groundwater level decline observed from 2008 to 2017 that proceeds at the same rate until 2040. Pauloo et al.^13^ generated three BAU scenarios that correspond to hydrologic variability from 1998–2017, 2003–2017, and 2008–2017. We use the final scenario (2008–2017) because it represents the hydrologic period of greatest decline, and may be interpreted as intensive groundwater use characterized by relatively dry years compared to a historical average.

**Figure 2:**
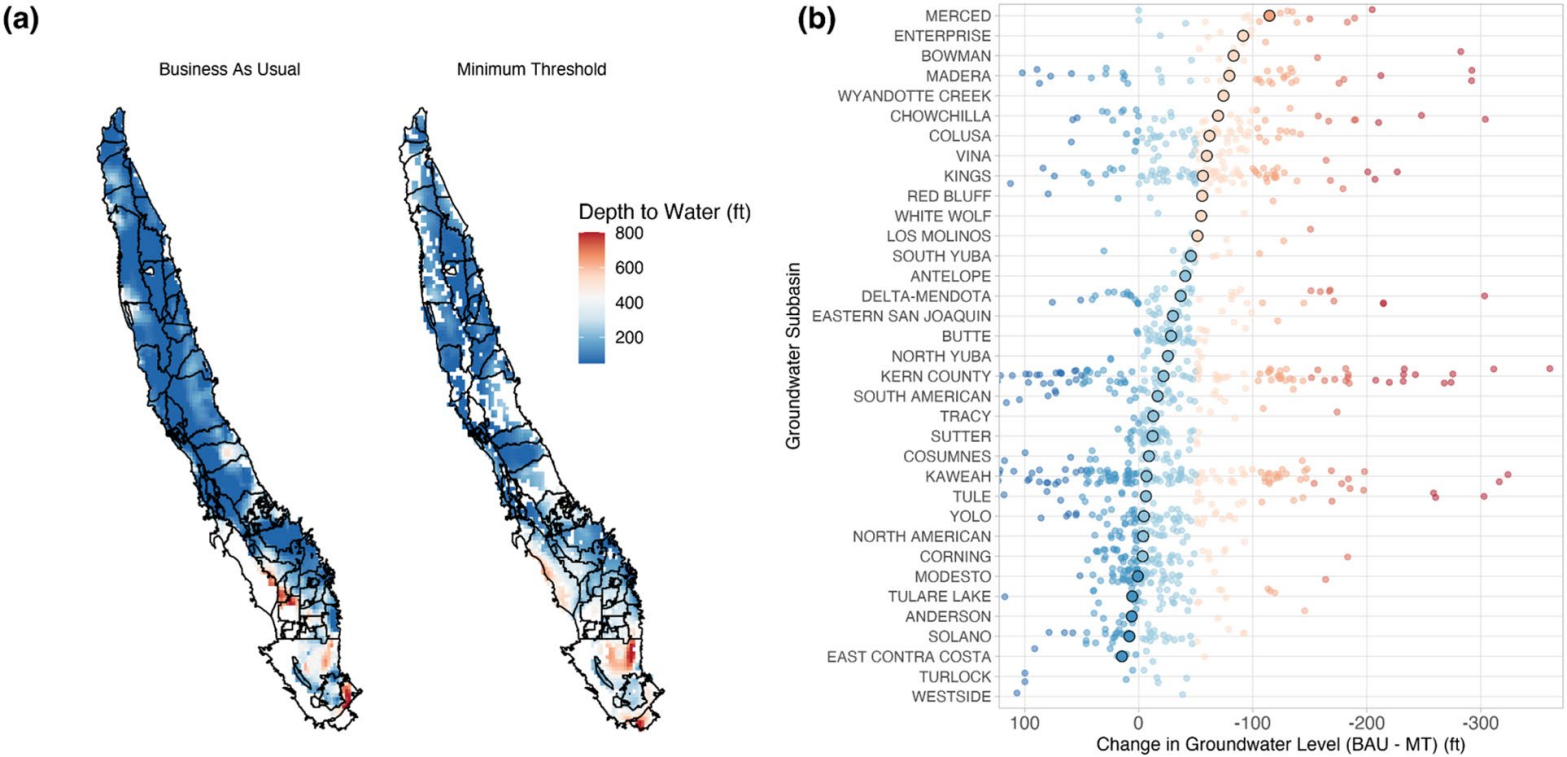
Difference between Business As Usual and proposed Minimum Thresholds. (**a**) Groundwater level spatial patterns for BAU and MT scenarios and (**b**) per-monitoring well groundwater level differences suggest that groundwater level MTs are generally deeper than BAU levels. In (**b**), negative, red values represent locations where the MT is *deeper* than the modeled BAU elevation (accelerated depletion), and positive, blue values represent locations where MTs are above the BAU elevation (protective sustainability criteria). In addition, darkly circled points represent the median difference in BAU and MT elevations for each GSP.

MTs higher than BAU levels suggest sustainability criteria protective of BAU decline, whereas MTs below BAU levels suggest sustainability criteria that permit accelerated depletion. MTs close to BAU levels indicate parity between sustainability criteria and BAU decline. We find approximate parity between MTs and BAU levels in most subbasins (21 out of 35), where the median MT is within 50 feet of estimated BAU groundwater levels. Protective sustainability criteria are observed in 6 of 35 subbasins, which have median MTs greater than 50 feet above than BAU levels. Finally, sustainability criteria that permit accelerated depletion are observed in 8 of 35 subbasins, where the median MTs are more than 50 feet deeper than BAU levels. Although groundwater levels will not be allowed to decline below MTs after 2040, it is striking that MTs so closely resemble BAU groundwater decline, which as we demonstrate in the following section, would lead to thousands of dry wells by 2040 if these sustainability thresholds are reached (Fig. 2).

### Thousands of domestic and public supply wells are at risk under proposed minimum thresholds (MTs)

**Figure 3:**
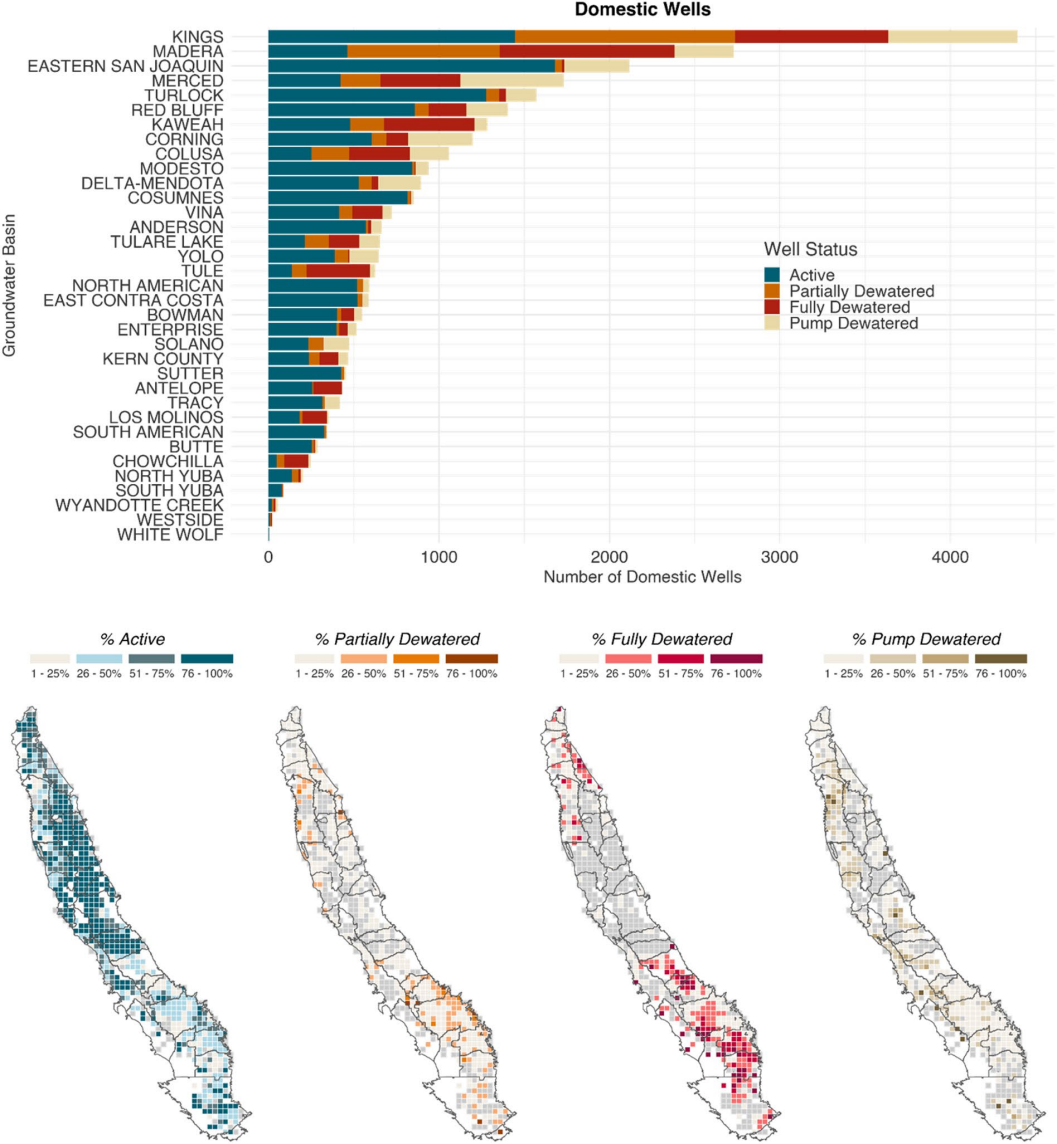

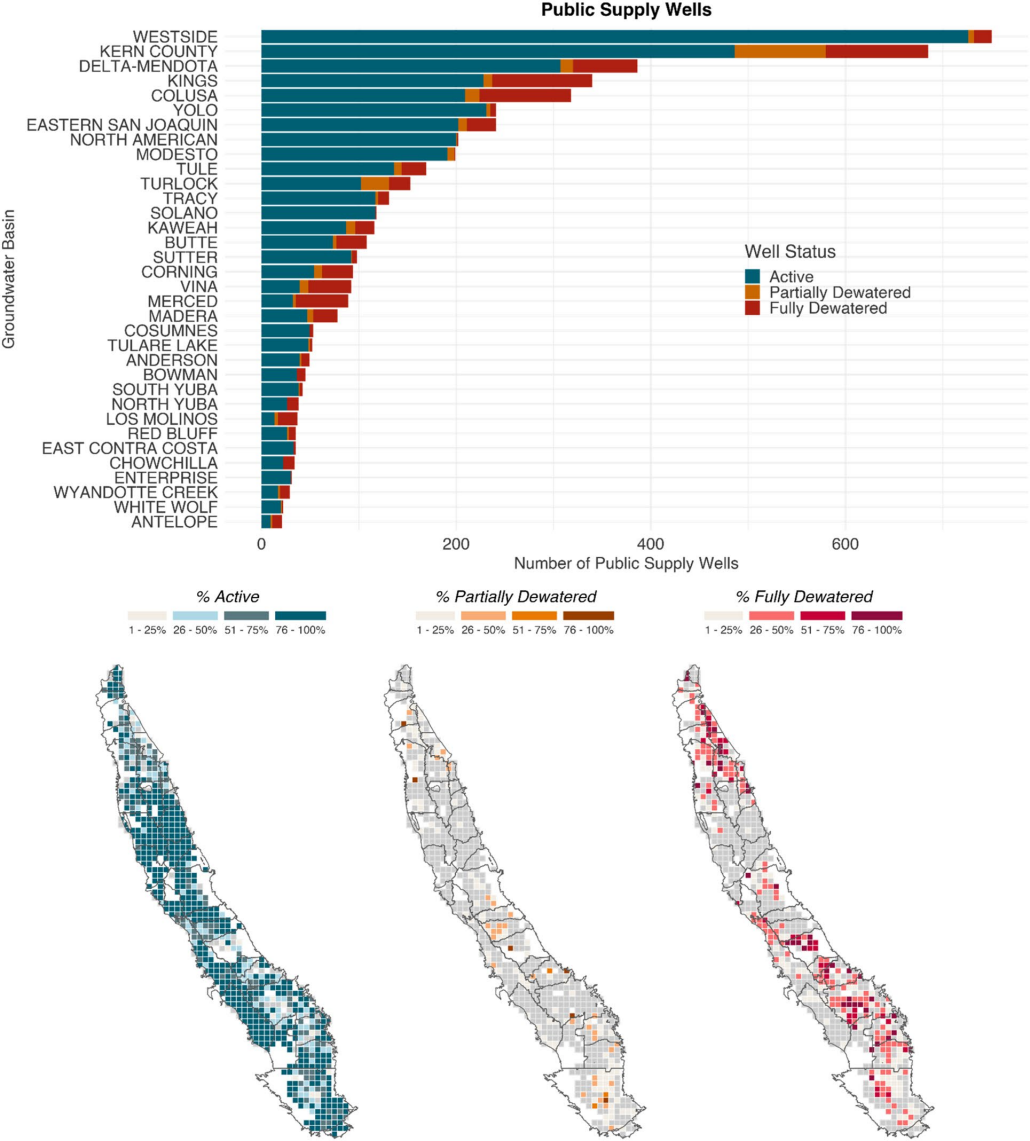
Domestic and public supply well status by groundwater subbasin. Barcharts represent the count of wells per subbasin by status (active, partially dewatered, fully dewatered, pump dewatered). Maps show the percentage of fully and partially dewatered domestic and public supply wells, by township and subbasin. Domestic and public supply well vulnerabilities tend to colocate.

Using the proposed MTs throughout the study area, we analyzed and mapped the distribution of domestic and public supply well failure (Fig. 3). Failure is defined in three ways, arranged here in decreasing severity: (1) wells are fully dewatered when the MT is set below the total completed depth of the well; (2) submerged pumps are dewatered when the MT drops below the pump depth; and (3) wells are partially dewatered when the MT drops below the top of the well screen. Information on public supply well pump depths is unavailable, but may be estimated for domestic wells, thus for case 2 above, only domestic well pump vulnerability was evaluated.

Across the entire study area, about 30,000 domestic and 5300 public supply wells had sufficient information for analysis. Of these wells, approximately 18% of domestic wells and 15% of public supply wells (Fig. 3) would be fully dewatered under proposed MTs. An additional 14% of domestic wells and 6% of public supply wells would be partially dewatered under proposed MTs. In total, we estimate that 32% of domestic wells (n = 9285) and 21% of public supply wells (n = 1168), or 30% of drinking groundwater wells would be partially or fully dewatered if groundwater levels reach MTs. An additional 16% of domestic wells have proposed MT groundwater levels below estimated pump locations^22^ (Supplementary Table 1). This indicates that over ten thousand drinking water wells are impacted by MTs set by GSPs, potentially threatening household and municipal water security.

Furthermore, where higher concentrations of domestic wells are vulnerable, higher concentrations of public supply wells are also vulnerable. While high percentages of wells are predicted to be impacted in areas that saw many well failures during the 2020–2022 drought, areas in the northern Central Valley with comparably high groundwater levels are also likely to incur wells failures if MTs are reached.

Impacts to household water supplies are not spatially uniform. Rather, they vary by basin, depending on how the GSPs within the basin define sustainability. When aggregated to the groundwater basin level, 3–80% of domestic wells, and 0–65% of public supply wells are vulnerable, respectively (see Fig. 3 and Supplementary Table 2 for a complete view of impacted wells by GSP).

### Monitoring networks cover most drinking water wells

**Figure 4:**
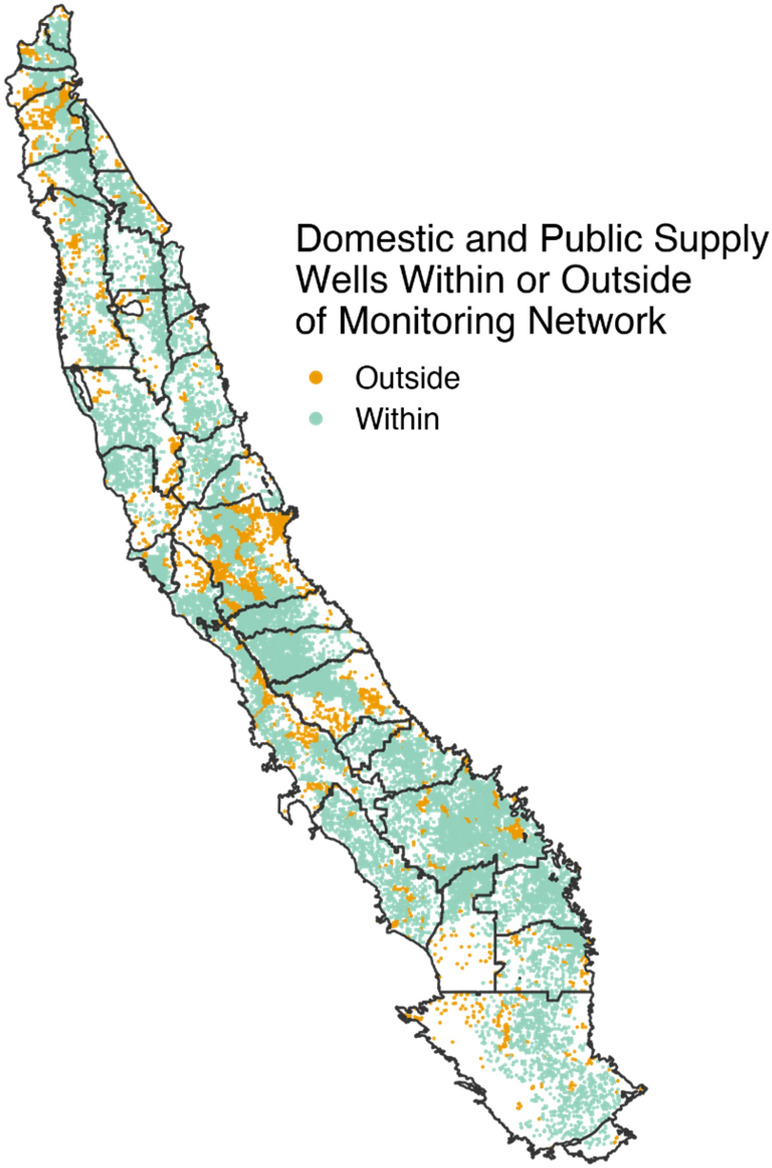
Monitoring network coverage of wells. Each point represents a drinking water well (domestic or public supply), and color denotes whether the well falls within (green) or outside (orange) proposed monitoring networks. Eastern San Joaquin and Red Bluff cover the lowest percent of domestic and public supply wells, at 54% and 59% coverage, respectively.

The above analyses only include wells located within locally-defined monitoring networks across the study area. This is because MTs are set at monitoring wells and are therefore locally specific. For wells located outside of monitoring network coverage, GSPs provide no quantified sustainability criteria. In other words, groundwater conditions in these areas fall outside the scope of locally-defined sustainability. These wells are also excluded from groundwater level monitoring because they fall outside the reasonable lateral extent that interpolation between monitoring wells provides. As a result, adverse effects on these wells caused by declining groundwater levels may go undetected. On average, we find that 90% of drinking water wells fall within monitoring networks (Fig. 4). However, 8 GSPs cover less than 80% of the drinking water wells within groundwater basin boundaries, including Eastern San Joaquin, which covers the smallest fraction of wells (54%) in the groundwater basin.

### Disadvantaged communities are likely to be impacted

Like most environmental hazards, drinking water impacts are not evenly distributed across the population at large but tend to be concentrated among disadvantaged communities (DACs). To evaluate the potential for disproportionate impacts from GSPs, we evaluate impact to DACs that, in California’s Central Valley, often rely on shallow wells and are among the most sensitive water users to existing drinking water inequities^13,14^. In the study area, we identified 193 DACs, which are communities “with an annual median household income (MHI) less than 80 percent of the statewide annual MHI”^26^, amounting to a total population of about 2.8 million people. Only 4 out of 35 subbasins evaluated have no DACs within their boundaries.

Of the 3484 domestic and public supply wells located inside DAC boundaries, representing 10% of domestic and public supply wells available for analysis in the Central Valley, 79% (n = 2752) of wells in DAC boundaries fall within monitoring networks and have sufficient construction information to be evaluated for failure under GSP conditions. Of the 2752 wells available for analysis, nearly 40% (n = 1051) are vulnerable to MT groundwater levels. Drinking water wells in DACs are impacted at 43%, about the same rate as those that fall outside of DACs, which indicates that DACs have a similar probability of losing drinking water wells as non-DACs. However, the lower MHI in DACs may result in fewer financial and political resources to restore access.

**Figure 5:**
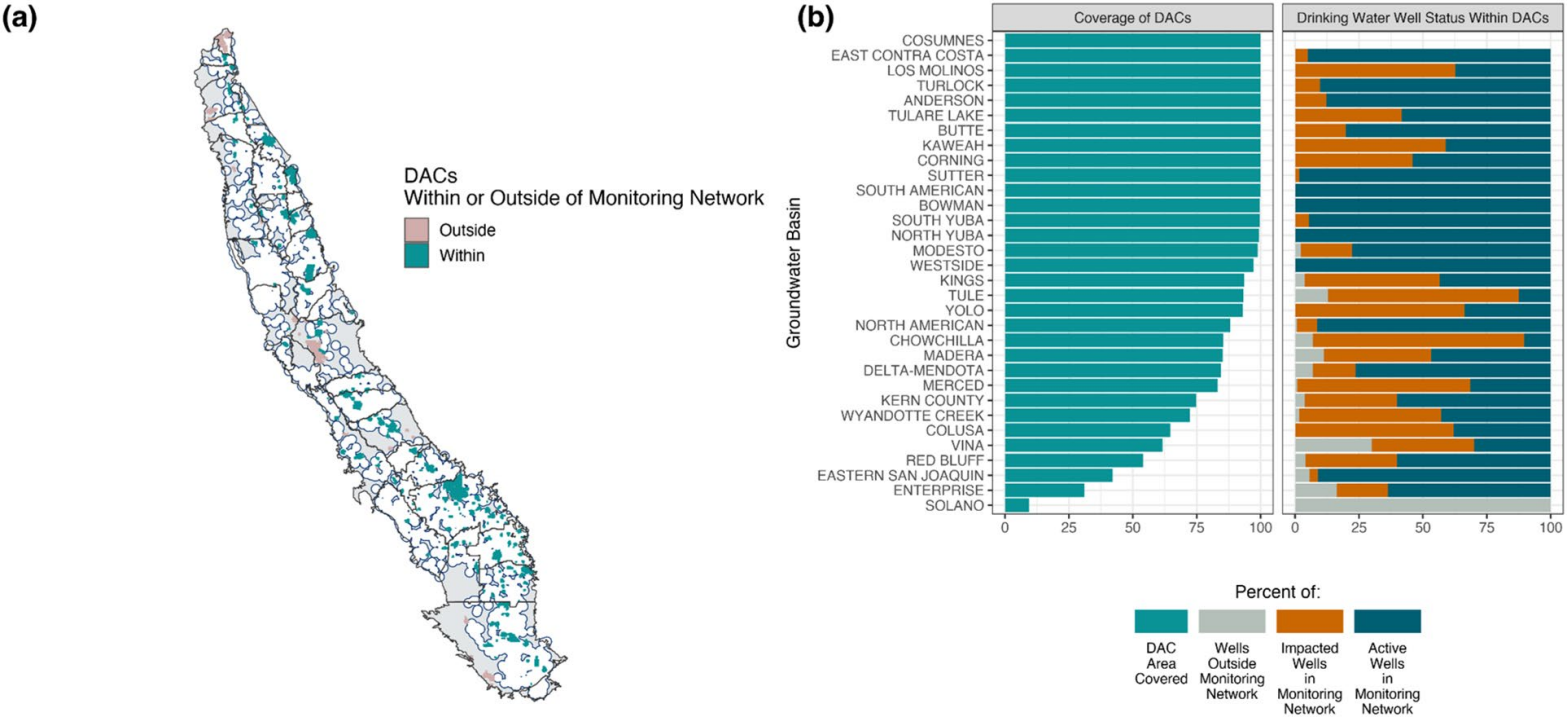
Coverage of domestic wells and impacts to wells in DACs. (**a**) Area of DACs covered (green) by the monitoring network (white) and (**b**) percentage of DAC surface area within and outside of the monitoring network, and drinking water wells impact within DACs. Solano, Enterprise, Eastern San Joaquin, and Red Bluff cover the fewest DACs, covering 0%, 33%, 50%, and 60% of their DACs, respectively. Cosumnes contains DACs, but there are no drinking water wells within the Cosumnes DACs.

We also find that 55% of DACs (n = 106) within groundwater basin boundaries are fully represented in monitoring networks (> 90% of area covered). Furthermore, 29% of DACs (n = 55) are partially covered (50–89% of area covered). However, 31 DACs in 11 GSPs have at least 50% of their area outside of GSP monitoring networks (Fig. 5). Finally, nearly 15% of domestic and public supply wells that fall within DAC boundaries (n = 508) are entirely outside of monitoring networks.

## Discussion

California’s groundwater reform, SGMA, shows that locally-determined, quantitative sustainability criteria are not necessarily sustainable for all resource users. Local definitions of sustainability, namely minimum thresholds (MTs), generated a wide distribution of impacts. The majority of subbasins (21 out of 35) have MTs that specifically accommodate ongoing groundwater level decline^27^ (Figs. 1, 2). This allows some groundwater users to continue to extract groundwater resources, but, if followed, also holds the potential to dewater up to 30% of groundwater wells used for drinking water (Fig. 3), an outcome with considerable public health and wellness consequences, particularly for low-income and already environmentally burdened residents (Fig. 5). Furthermore, 10% of drinking water wells and 16% of DACs fall outside the purview of monitoring networks, leading to increased uncertainty about the applicability of MTs in these areas, as well as hindering the ability of agencies and stakeholders to comprehensively understand local groundwater conditions (Figs. 4, 5).

These findings highlight the importance of sustainability definitions, such as MTs, in shaping governance outcomes. Furthermore, they underscore the need to thoughtfully engage in discussing and evaluating locally-defined sustainability goals and their implementation. In this discussion we first explore the implications of the variation in sustainability definitions documented for groundwater management, water security, and environmental equity. Subsequently we summarize policy implications and recommendations, and consider practical guidelines to strengthen on-going groundwater management efforts.

### Implications for groundwater management

Like many food-producing regions worldwide, California is heavily dependent on groundwater pumping for food production, and for domestic and municipal drinking water supply. Increased reliance on groundwater for irrigated agriculture, particularly during drought to supplement lost surface water supply, drives groundwater depletion^11^. However, depletion does not affect all resource users equally; deeper wells are required to access retreating groundwater levels, which in turn requires capital. SGMA aimed to address the issue of groundwater sustainability and the protection of all beneficial users of groundwater by combining a top-down and bottom-up policy strategy. The state provided local actors with a flexible framework to self-organize into agencies and design sustainability plans that balance agricultural water needs with other uses (e.g., domestic, municipal, or ecosystem users), while still allowing for central government intervention^24^.

However, as this paper has shown, SGMA is likely to have mixed effects. In total, 36 GSPs set sustainability criteria consistent with business as usual groundwater depletion through the end of the implementation period, which does not indicate a gradual deceleration of groundwater use ending in sustainable yield (Figs. 1, 2). Furthermore, Pauloo et al.^13^ modeled a business as usual groundwater level decline to the year 2040 and projected 5966–10,466 domestic well failures in the Central Valley. In this study, we estimate 9285 domestic wells would fail if MTs are reached (Fig. 3), which implies that proposed sustainability criteria are consistent with the projected range of business as usual groundwater overdraft.

Although continued depletion through the end of SGMA’s implementation period may provide a cushion for agricultural economies to transition to more sustainable rules around extraction, it remains unclear how extraction rates will slow down when that period ends. For example, many plans do not address demand-side solutions, such as agricultural transitions to less water-intensive crops, land fallowing^28^, or pumping reductions^29^. Instead, most focus on supply-side solutions, like managed aquifer recharge (MAR), which require wet winters and infrastructure to divert and recharge groundwater. In other words, many GSAs have elected to avoid or delay alternative groundwater solutions to reduce groundwater over-extraction, and therefore depletion, with consequences for shallow wells. SGMA attempts to balance the needs of various groundwater users by empowering local agencies to define and implement sustainable groundwater use. However, the effectiveness of the sustainability criteria that have been proposed in GSPs, and their ability to mitigate depletion and detrimental impacts to vulnerable users, remains uncertain.

### Implications for water security and environmental equity

Water insufficiency and inaccessibility undermines water security^30^; this in turn negatively affects food security^31^, human livelihoods^32^, and health and wellbeing^33^. While people across the world are impacted by water insecurity, vulnerable, disadvantaged communities often bear the brunt of the impacts. As stated above, DACs in the Central Valley are overwhelmingly groundwater dependent and typically rely on shallow wells for drinking water^15^. During the 2012–2016 drought, more than 2,000 groundwater-wells went dry in the study area^34^, with impacts concentrated in Latino and low-income communities^35^. During this crisis, the California government deployed an emergency household program that proved cost-prohibitive and unsustainable as an ongoing disaster assistance solution^36^.

Decades of groundwater overdraft in California's Central Valley have lowered groundwater levels near well screens and hence, even small fluctuations in groundwater levels during periods of average decline, or droughts that increase reliance on pumped groundwater, can be enough to cause thousands of wells to run dry. GSP-established MTs, theoretically, should prevent future need for state support of dry domestic wells when groundwater levels decline. For instance, GSAs are encouraged to outline the impacts of MTs on groundwater users and groundwater levels should not dramatically exceed MTs in extreme drought conditions.

**Figure 6:**
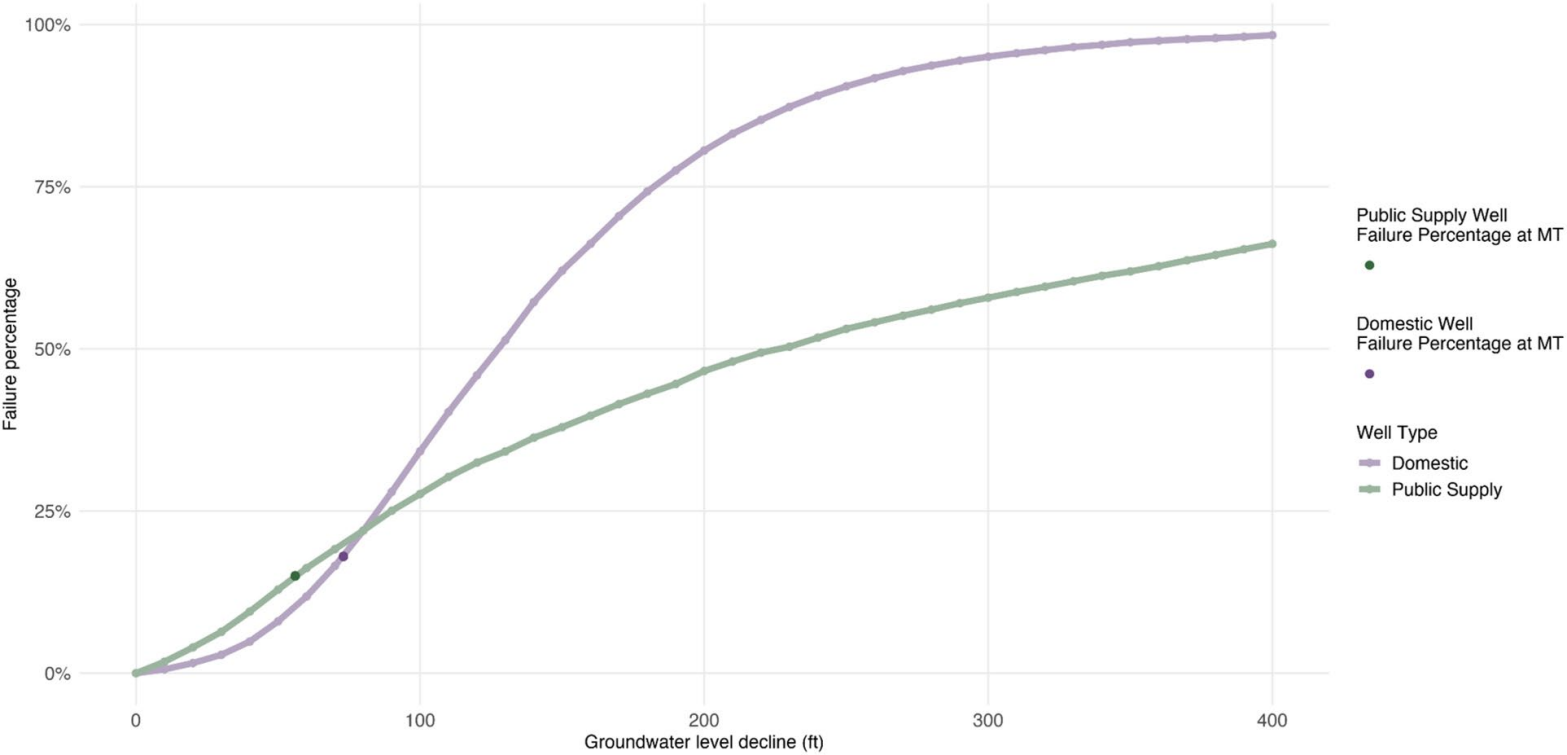
Percentage of wells fully dewatered by feet of groundwater level decline. Lines represent the percentage of public supply (green) or domestic (purple) wells that would be fully dewatered (total completed depth above groundwater level) at different levels of uniform groundwater level decline from the 2019 baseline. Darker points represent the percent of domestic (18%) and public supply wells (15%) that fail at MT groundwater levels.

Yet, few GSPs have examined the tradeoffs between MTs and well failure. As a result, communities with MT groundwater levels below well depths may, once again, lose access to drinking water and likely face steep and unaffordable costs to construct deeper wells. Furthermore, we find that GSPs did not completely include vulnerable communities or domestic and public supply wells in monitoring networks used to define sustainability targets (Figs. 5, 6). In other words, if groundwater levels drop near shallow wells in DACs that fall outside of monitoring networks, GSAs may not have defined sustainability criteria nor monitoring wells to proactively address potential detrimental impacts to drinking water. Two factors explain, in part, gaps in monitoring network coverage: residents of DACs were rarely included in the development of GSPs and DACs have little formal representation in GSA governance^37^. For example, 84% of DACs across California have no formal representation in their GSA^38^.

GSPs that fail to cover DACs with monitoring networks and that lack formal DAC representation in GSP governance run the risk of perpetuating systematic inequality without viable mechanisms to detect and remediate harm. SGMA designated existing public agencies “with water supply, water management, or land use responsibilities within a groundwater basin”^25^—often agricultural and urban surface water districts—as the GSAs, the implementation vehicles for the reform. This design of SGMA empowered local surface water users who have historically controlled water rights and infrastructure, likely resulting in diminished access (and therefore diminished input) for groundwater-dependent users, such as farmers without district affiliations, tribes, and unincorporated disadvantaged communities^39,40^.

### Policy implications and recommendations

Our results suggest that broad definitions of sustainability, deployed through a polycentric environmental governance regime, may not result in sustainable outcomes for groundwater users in general and especially for vulnerable resource users. Local agencies have set a variety of sustainability criteria, namely minimum thresholds (MTs), presenting regional differences in the approach taken, and overwhelmingly leaning towards status-quo, business-as-usual targets that support continued groundwater depletion. If sustainability reforms result in detrimental outcomes for vulnerable resource users, even under legally and legitimate local processes, and under the guidance of experts, of state-led technical assistance, and of knowledgeable local actors, what measures can be taken to improve local sustainability criteria and reduce negative impacts to vulnerable resource users?

First, clarifying the implications sustainability definitions, especially impacts to vulnerable populations and underrepresented groups, may create more transparency and discussion around these decisions. While these definitions, such as MTs, may not be set in statute, state agencies charged with implementation should consider the use of explicit, minimally acceptable definitions of sustainability. For example, state agencies might lead with more clearly defined success metrics for sustainability criteria (e.g., no more than 10% of active wells are dewatered under sustainability criteria).

Second, ideally, each GSA would map and understand the physical and economic tradeoffs between stringent sustainability metrics and impacts to drinking water wells. To illustrate the physical tradeoff between stricter MTs and drinking water well failure rates, we estimate the impacts of incremental groundwater level decline on domestic and public supply wells (Fig. 6), noting that stricter MTs would shorten the glide path for existing agricultural users to transition from depletive to sustainable groundwater management. Across the entire study site, nearly all domestic wells are fully dewatered at 350 feet of groundwater level decline from CGWL while about 70% of public supply wells are fully dewatered at the same level.

Meaningful benefits to well security may be realized at even marginally stricter MTs. For example, across the entire Central Valley, if decline was limited to 70 feet instead of 80 feet, it would reduce the percent of domestic wells that are fully dewatered by 5% (1395 wells) and public supply wells by 3% (154 wells). Well failures, left unaccounted for, are unknown externalities on the path to purported sustainability. Predicting well failures allows decision-makers to internalize well failure into the cost of sustainable management criteria (i.e., MTs), and permits an evaluation of the economic and ethical tradeoffs between well failure and lost groundwater for other extractive beneficial uses such as agricultural, industrial, and municipal consumption. Furthermore, state and local policies might also explicitly consider underrepresented groups in well failure predictions (e.g., well impact analyses are required to inform financial planning and development of mitigation funds for impacted households).

Finally, regulators can and should continue to develop, test, validate, share, and apply tools to provide unequivocal success criteria to local entities, and to confidently enable auditing of local plans against these criteria. It is not uncommon for states to publish natural resource technical studies (e.g., C2VSim^41^, IWFM^42^, SGMA Best Management Practices^25^), develop and release computer modeling tools and frameworks, and offer technical assistance and educational programs. However, as well executed these initiatives may be, modeling tools are biased to information available at the time of development or data publishing, which underscores a need for continuous and adaptive science, policy, outreach, and ultimately, resource management.

Groundwater sustainability agencies, sustainability plans, and the broader effort to reform groundwater management in California have wide reaching implications for beneficial uses including drinking water. While variable, most local responses have the potential to harm disadvantaged and underrepresented communities and allow for the persistence—and perhaps enablement—of some of the unsustainable conditions that SGMA initially set out to end. By evaluating the impact of current plans on vulnerable resource users, local agencies and regulators can pave the way for a more just and sustainable future of groundwater use.

## Methods

### Groundwater sustainability data collection

A total of 60 GSPs covering the Central Valley aquifer system were compiled and reviewed to extract MTs reported at representative monitoring wells. These GSPs were chosen because they are part of the largest contiguous aquifer system in California^43^, and one with the most severe groundwater depletion; most critically overdrafted basins are in the Central Valley^44^. Furthermore, although natural hydraulic gradients have an impact in some areas of the Central Valley aquifer system, notably on the edges of the valley subject to mountain front recharge, much of the aquifer is so heavily developed that groundwater flow directions are principally dictated by local flow fields induced by pumping and irrigation recharge—the largest parts of the region's groundwater budget. Thus, changes in management of the Central Valley aquifer will reasonably impart substantial impact on the region’s hydrology and groundwater users^45–47^.

We used the well completion reports database by Pauloo et al.^13^, who compiled 943,469 wells from the California Department of Water Resources (CA-DWR)^22,48^, and seasonal groundwater level measurements from the year 2019 in the CA-DWR periodic groundwater level measurement database^49^.

Each MT groundwater well reported in the GSPs was evaluated by: (i) identifying well coordinates, MTs, and reference ground surface elevations in the California Department of Water Resources Water Data Library (n = 1403), (ii) georeferencing wells if no coordinates were available (n = 45), and (iii) when wells were absent from the Water Data library, we used well reference elevation estimates from nearby wells with known ground surface elevations (n = 26). We performed quality-control measures on a total of 1474 MT wells, homogenizing units to compare water levels set by the MT wells to the groundwater-pump depth intakes of domestic wells in units of Depth Below Ground Surface (DBGS). We used the equation DBGS = Reference elevation − Feet above mean sea level (MSL) to convert units reported in feet above MSL to DBGS. We also filtered domestic and public supply wells in well completion reports that had: (i) elevations below current groundwater levels, (ii) interpolation boundaries included (to perform analysis), and (iii) been constructed after 1990 to conservatively include only operational wells^27,50^ (n = 9488 wells were filtered out of the analysis). After application of the selection criteria described above, we arrived at 29,567 domestic and 5259 public supply wells for analysis.

### Comparing current and business as usual groundwater levels versus proposed levels in GSPs

California’s Central Valley is a clastic sedimentary alluvial aquifer-aquitard complex wherein variability in the hydraulic conductivity of sediments can range over 4–7 orders of magnitude^51,52^ and operate in a semi-confined manner. For this reason, we used "groundwater level" rather than "potentiometric surface" which would describe a pressurized confined aquifer, or "water table" which would describe an unconfined system.

To compare existing groundwater level conditions and proposed conditions under plans, we generated two spatial layers. We used the average 2019 groundwater levels based on seasonal (spring and fall) groundwater level measurements as the current groundwater elevation of the aquifer system. We selected 2019 as a reference year because it was not a dry year, which allowed for estimated groundwater levels that reflect average, non-drought conditions. We examined reported MTs at representative monitoring well points in 60 GSPs totaling 1474 unique monitoring wells with specified minimum (elevation) thresholds as the outcome-condition if GSPs were implemented. We log-transformed both elevation measurements (i.e., 2019 and MT groundwater elevations) and applied ordinary kriging to normalize the data distribution and control outliers^53,54^. Since the expected value of transformed-log kriging estimates is not equal to the sample mean, we fitted an exponential model to correct estimates:

$$g={k}_{0}\times exp\left[ln\left({\widehat{g}}_{0K}\right)+ \frac{{{\sigma }^{2}}_{0K}}{2}\right].$$

In this equation, g is the corrected log-transformed groundwater level, ${\widehat{g}}_{0K}$ is the ordinary kriging estimate, ${{\sigma }^{2}}_{0K}$ is the kriging variance, and ${k}_{0}$ is the correction factor, proportional to the ratio of the mean of the sample values to the mean of the log-transformed kriging estimates. We compared original MT values to mean corrected values and found no more than a 5% difference. Further, we also calculated the 95% confidence interval of the kriging estimates to measure uncertainty associated with the kriging estimate. We then intersected the 1474 MT wells with the 2019 raster and then plotted the difference between the current groundwater level and MT levels to visualize elevation differences (Fig. 1).

Next, the estimated MT groundwater surface was compared to a modeled BAU groundwater level from Pauloo et al.^13^, based on groundwater level decline observed from 2008 to 2017 that proceeds at the same rate until 2040. Pauloo et al.^13^ generate three BAU scenarios that correspond to hydrologic variability from 1998–2017, 2003–2017, and 2008–2017. We used the final scenario (2008–2017) because of the three, it represents the hydrologic period of greatest decline, and may be interpreted as intensive groundwater use characterized by relatively dry years compared to a historical average. We plotted both modeled groundwater level surfaces next to one another (Fig. 2a) to show the difference between MT and BAU water levels at each MT monitoring well location (Fig. 2b). We then intersected the MT well locations with the BAU raster, subtracted the MT groundwater level from the BAU groundwater level at each monitoring well location, and calculated the median difference from all monitoring wells within a subbasin (Fig. 2b).

### Dry well analysis

To calculate the number of domestic and public supply wells vulnerable to failure under proposed GSPs, we assumed wells fail when the MT drops below the total depth of the well. In other words, when the water level is below the bottom of the well. This is a conservative estimate given that wells dewater and experience mechanical damages when groundwater levels approach pump levels, and cavitation occurs. Applying this assumption to the data, we distinguished between *active* wells where the top of well screens are deeper than groundwater levels, *partially dewatered wells* with the top of the well screen shallower than groundwater levels, and *fully dewatered wells* where total completed depth is shallower than the MT groundwater level. For domestic wells, we also calculated the vulnerability of submerged pumps. If the water level drops below the estimated pump depth of the well, we consider the *pump dewatered*. We mapped the spatial distribution of failing wells, showing percentage ranges of well failure by approximate township grids (Fig. 3).

In addition, we defined a set of 41 uniform groundwater level decline scenarios ranging from 0 to 400 feet, which we subtract from the 2019 groundwater level (Fig. 6). The uniform decline scenarios are used to evaluate the well failure that would result from the given amount of groundwater level decline. The rate of fully dewatered wells per uniform decline scenario is calculated by comparing the total completed depth of each well to the estimated groundwater level.

### Coverage of vulnerable communities in monitoring networks

Representative groundwater monitoring networks are mandated by SGMA and evaluated by the California Department of Water Resources during GSP review. The CA-DWR guidelines for monitoring networks suggests a range from 2 to 10 monitoring wells per 100 miles depending on the amount of pumping and complexity of the geology^55,56^ which implies that a monitoring well can represent 10–50 miles of lateral extent. To approximate this recommended coverage level, we defined 3.5 mile (radius) circular buffers around each monitoring well with the MT specified in GSPs (Supplementary Fig. S1). This was consistent with basins pumping more than 5000 acre-feet/year^57^. We compared the total area of the buffer to the area of the GSP to assess the percentage of monitoring network coverage. Moreover, we compared the buffer area to the location of domestic wells within a GSP to quantify the percentage of domestic wells that fall within the monitoring network.

While certain areas outside GSP monitoring networks may experience limited groundwater use, it is important to acknowledge that some regions, including those with domestic wells, lie beyond the scope of monitoring networks that can adequately represent groundwater conditions (Fig. 3). We assumed each monitoring well can reasonably depict groundwater conditions within a 3.5 mile radius around it, consistent with Best Management Practices from DWR^55^. The absence of monitoring wells does not necessarily imply groundwater overdraft, but it does present challenges in accurately measuring sustainable outcomes, particularly where domestic wells fall outside of the monitoring networks.

To compare monitoring network coverage of disadvantaged communities (DACs) we use a California Department of Water Resources (DWR) shapefile of the Census Designated Places considered DACs^58^. DACs are defined as communities “with an annual median household income (MHI) that is less than 80 percent of the Statewide annual MHI”.^26^ This shapefile included 576 DACs, which were then intersected with the GSP areas. When more than 50% of a DAC geometry fell within a GSP, it was included in the analysis. This resulted in 193 DACs. These DACs were then intersected with the monitoring network shapefile. The percent of DAC area covered by the monitoring network was calculated by dividing the DAC area within the monitoring network by the DAC area within the GSP area.

### Supplementary Information

Supplementary Information.

## Data Availability

The data that support the findings of this study are available in GSPCode at https://github.com/djbostic/GSPcode. These data were derived from the following resources available in the public domain: Domestic Wells—https://data.cnra.ca.gov/dataset/well-completion-reports and https://datadryad.org/stash/downloads/file_stream/213989. Groundwater Sustainability Plan Geometries—https://sgma.water.ca.gov/webgis/index.jsp?appid=gasmaster&rz=true. Periodic Groundwater Level Measurements—https://data.cnra.ca.gov/dataset/periodic-groundwater-level-measurements. Disadvantaged Communities—https://data.cnra.ca.gov/dataset/dacs-census. Business As Usual Groundwater Level Estimates—https://datadryad.org/stash/dataset/10.25338/B8Q31D.
