# Author Correction: Thousands of domestic and public supply wells face failure despite groundwater sustainability reform in California’s Central Valley

**DOI:** 10.1038/s41598-024-56006-4

**Published:** 2024-03-05

**Authors:** Darcy Bostic, Linda Mendez-Barrientos, Rich Pauloo, Kristin Dobbin, Victoria MacClements

**Affiliations:** 1 Rural Community Assistance Corporation, West Sacramento, USA; 2 Josef Korbel School of International Studies, University of Denver, Denver, USA; 3 Water Data Lab, Berkeley, USA; 4 Department of Environmental Science, Policy, and Management, University of California Berkeley, Berkeley, USA

Correction to: *Scientific Reports* 10.1038/s41598-023-41379-9, published online 08 September 2023

The original PDF version of this Article contained a repeated error, where the percentage of drinking groundwater wells was incorrectly given as 53%.

As a result, in the Results section, under the subheading ‘Thousands of domestic and public supply wells are at risk under proposed minimum thresholds (MTs)’,

“In total, we estimate that 32% of domestic wells (n = 9285) and 21% of public supply wells (n = 1168), or 53% of drinking groundwater wells would be partially or fully dewatered if groundwater levels reach MTs.”

now reads:

“In total, we estimate that 32% of domestic wells (n = 9285) and 21% of public supply wells (n = 1168), or 30% of drinking groundwater wells would be partially or fully dewatered if groundwater levels reach MTs.”

In addition, in the Discussion section,

“This allows some groundwater users to continue to extract groundwater resources, but, if followed, also holds the potential to dewater up to 53% of groundwater wells used for drinking water (Fig. 3), an outcome with considerable public health and wellness consequences, particularly for low-income and already environmentally burdened residents (Fig. 5).”

now reads:

“This allows some groundwater users to continue to extract groundwater resources, but, if followed, also holds the potential to dewater up to 30% of groundwater wells used for drinking water (Fig. 3), an outcome with considerable public health and wellness consequences, particularly for low-income and already environmentally burdened residents (Fig. 5).”

The original Article has been corrected.

