# Supplementary material for: Thousands of domestic and public supply wells face failure despite groundwater sustainability reform in California’s Central Valley
Source: Sci Rep. 2023 Sep 8;13:14797. doi: 10.1038/s41598-023-41379-9 (PMC10491797; doi:10.1038/s41598-023-41379-9)
Supplement: Supplementary file 1 — Supplementary Information. [file 41598_2023_41379_MOESM1_ESM.pdf]

Supplementary Table 1: Domestic Wells Impacted by GSP

GSP	Total Domestic Wells	Active Wells	Partially Dewatered	Fully Dewatered	Pump Dewatered
Aliso	2	0	2	0	0
Alpaugh	2	1	0	0	1
Anderson	665	570	14	16	65
Antelope	432	256	6	168	2
Bowman	548	400	27	74	47
Buena Vista	12	10	1	0	1
Butte	287	253	13	7	14
Central Kings	794	262	167	227	138
Chowchilla	247	48	45	140	14
Colusa	1058	252	220	355	231
Corning	1196	604	88	126	378
Cosumnes	851	816	16	4	15
Delano-Earlimart	27	8	1	17	1
East Contra Costa	586	521	27	3	35
East Kaweah	267	117	48	79	23
East Tule	413	92	51	258	12
Eastern San Joaquin	2115	1680	41	13	381
Enterprise	515	398	15	51	51
Farmers	2	2	0	0	0
Fresno County	15	5	4	3	3
Grassland	25	24	0	0	1
Gravelly Ford	6	4	0	0	2
Greater Kaweah	765	286	133	300	46
Henry Miller	2	0	2	0	0
James	33	11	5	3	14
Kern Groundwater Authority	303	139	38	102	24
Kern River	150	87	22	9	32
Kings River East	647	367	176	55	49
Los Molinos	353	183	17	142	11
Lower Tule River	84	18	14	42	10
Madera Subbasin Joint	2713	452	891	1026	344
McMullin Area	170	52	35	55	28
Merced	1730	421	234	471	604
Mid-Kaweah	250	76	18	151	5
Modesto	939	843	13	6	77
New Stone	1	0	1	0	0
North American	590	519	36	0	35
North Central Delta-Mendota	386	187	35	17	147
North Fork Kings	384	26	67	270	21
North Kings	2344	719	837	288	500
North Yuba	202	136	37	16	13
Pixley	81	15	19	42	5
Red Bluff	1403	858	81	222	242
Root Creek	8	6	1	0	1
San Joaquin River Exchange Contractors	463	311	34	19	99
Solano	472	234	86	1	151
South American	341	325	14	2	0
South Kings	19	9	4	0	6
South Yuba	87	78	5	3	1
Sutter	454	426	17	0	11
Tracy	418	316	13	0	89
Tri-County	18	3	0	14	1
Tulare Lake	653	212	142	177	122
Turlock	1571	1275	77	40	179
Vina	723	413	78	177	55
Westside	25	8	5	8	4
White Wolf	3	2	1	0	0
Wyandotte Creek	54	20	10	9	15
Yolo	645	387	81	6	171

Supplementary Table 2: Public Supply Wells Impacted by GSP

GSP	Total Public Supply Wells	Active Wells	Partially Dewatered	Fully Dewatered
Aliso	2	0	2	0
Alpaugh	2	1	0	0
Anderson	665	570	14	16
Antelope	432	256	6	168
Bowman	548	400	27	74
Buena Vista	12	10	1	0
Butte	287	253	13	7
Central Kings	794	262	167	227
Chowchilla	247	48	45	140
Colusa	1058	252	220	355
Corning	1196	604	88	126
Cosumnes	851	816	16	4
Delano-Earlimart	27	8	1	17
East Contra Costa	586	521	27	3
East Kaweah	267	117	48	79
East Tule	413	92	51	258
Eastern San Joaquin	2115	1680	41	13
Enterprise	515	398	15	51
Farmers	2	2	0	0
Fresno County	15	5	4	3
Grassland	25	24	0	0
Gravelly Ford	6	4	0	0
Greater Kaweah	765	286	133	300
Henry Miller	2	0	2	0
James	33	11	5	3
Kern Groundwater Authority	303	139	38	102
Kern River	150	87	22	9
Kings River East	647	367	176	55
Los Molinos	353	183	17	142
Lower Tule River	84	18	14	42
Madera Subbasin Joint	2713	452	891	1026
McMullin Area	170	52	35	55
Merced	1730	421	234	471
Mid-Kaweah	250	76	18	151
Modesto	939	843	13	6
New Stone	1	0	1	0
North American	590	519	36	0
North Central Delta-Mendota	386	187	35	17
North Fork Kings	384	26	67	270
North Kings	2344	719	837	288
North Yuba	202	136	37	16
Pixley	81	15	19	42
Red Bluff	1403	858	81	222
Root Creek	8	6	1	0
San Joaquin River Exchange Contractors	463	311	34	19
Solano	472	234	86	1
South American	341	325	14	2
South Kings	19	9	4	0
South Yuba	87	78	5	3
Sutter	454	426	17	0
Tracy	418	316	13	0
Tri-County	18	3	0	14
Tulare Lake	653	212	142	177
Turlock	1571	1275	77	40
Vina	723	413	78	177
Westside	25	8	5	8
White Wolf	3	2	1	0
Wyandotte Creek	54	20	10	9
Yolo	645	387	81	6
